# Cancer DNA vaccines: current preclinical and clinical developments and future perspectives

**DOI:** 10.1186/s13046-019-1154-7

**Published:** 2019-04-05

**Authors:** Alessandra Lopes, Gaëlle Vandermeulen, Véronique Préat

**Affiliations:** Université Catholique de Louvain, Louvain Drug Research Institute, Advanced Drug Delivery and Biomaterials, Avenue Mounier, 73, B1.73.12, B-1200 Brussels, Belgium

**Keywords:** DNA vaccines, Cancer, Combination therapy, Immunotherapy, Immuno-oncology, Cancer vaccines, Antigens, Neoantigens

## Abstract

The recent developments in immuno-oncology have opened an unprecedented avenue for the emergence of vaccine strategies. Therapeutic DNA cancer vaccines are now considered a very promising strategy to activate the immune system against cancer. In the past, several clinical trials using plasmid DNA vaccines demonstrated a good safety profile and the activation of a broad and specific immune response. However, these vaccines often demonstrated only modest therapeutic effects in clinical trials due to the immunosuppressive mechanisms developed by the tumor. To enhance the vaccine-induced immune response and the treatment efficacy, DNA vaccines could be improved by using two different strategies. The first is to increase their immunogenicity by selecting and optimizing the best antigen(s) to be inserted into the plasmid DNA. The second strategy is to combine DNA vaccines with other complementary therapies that could improve their activity by attenuating immunosuppression in the tumor microenvironment or by increasing the activity/number of immune cells. A growing number of preclinical and clinical studies are adopting these two strategies to better exploit the potential of DNA vaccination. In this review, we analyze the last 5-year preclinical studies and 10-year clinical trials using plasmid DNA vaccines for cancer therapy. We also investigate the strategies that are being developed to overcome the limitations in cancer DNA vaccination, revisiting the rationale for different combinations of therapy and the different possibilities in antigen choice. Finally, we highlight the most promising developments and critical points that need to be addressed to move towards the approval of therapeutic cancer DNA vaccines as part of the standard of cancer care in the future.

## Background

Over the last few years, immunotherapy has received increasing attention as a strategy for cancer treatment, and many different approaches are being developed to improve the clinical outcome in cancer patients [1]. The main types of immunotherapy now being used to treat cancer include (i) monoclonal antibodies against specific antigens [2], (ii) immune checkpoint blockade (ICB) to release the “breaks” of T cells [3, 4], (iii) chimeric antigen receptor (CAR) T cell therapy, using a patient’s autologous cells [5], (iv) oncolytic viruses that selectively kill cancer cells and (v) cancer vaccines [6–8]. Currently, a few immunotherapeutic treatments are commercially available, such as anti-CTLA4, anti-PD1 and anti-PD-L1, CAR T cells against acute lymphoblastic leukemia and B-cell lymphoma, among others. Despite their costs and their immune-related side effects, their success has aroused interest in cancer immunotherapy as a new therapeutic option for cancer patients.

Cancer vaccines represent a promising strategy to induce a specific and long-lasting immune response against tumor antigens (TAs). TAs are mainly proteins overexpressed in the tumor tissue that play a central role in tumor initiation, progression and metastasis [9, 10]. Since the characterization of the first tumor antigen, the melanoma antigen (MAGE) in 1991 [11], a growing number of TAs have been identified. TAs can be classified into 2 main types (Table 1):

- *Mutational antigens*. These are derived from mutated self-proteins, which should not be present in normal cells. Some of these genes may be directly related to cancer development (oncogenes and tumor suppressor genes, such as Ras and Bcr-Abl) [12]. In many papers, these antigens are called “tumor-specific antigens” (TSAs). However, this specificity is relative because they can potentially be found in other tumors or even in any altered but nonmalignant cells [13]. Other unique TAs may have or not an association with tumor progression and are the result of the genetic instability of cancer cells. These are classically called “neoantigens”.
- *Tumor-associated antigens (TAAs).* These are nonmutated proteins overexpressed or aberrantly expressed in cancer cells [13, 14]. They include products of silent genes, such as oncofetal or cancer/testis antigens, which are not expressed in postnatal tissues or are normally expressed only in placenta and testis; differentiation antigens, which are tissue-specific proteins overexpressed in cancer cells; and universal tumor antigens, which are expressed in low amounts in normal tissues, but overexpressed in cancer [13].
  In the category of TAAs, we can include the oncoviral TAAs, which are non-self TAs and non-human proteins, expressed only by malignant cells transformed after an infection by an oncogenic virus. Examples of oncogenic viruses are human papilloma virus for cervical cancer and Epstein-Barr virus for nasopharyngeal carcinoma [15].

**Table 1:** Categories of TAs

TA category	TA subtype	Examples
Mutational antigens	Products of mutated oncogenes (TSA)	P53, Ras, Bcr-Abl
	Neoantigens	Case-specific mutations
Tumor associated antigens (TAAs)	Products of silent genes	Cancer/testis antigens (a-fetoprotein, MAGE-1, NY-ESO1)
	Differentiation antigens	Gp100, tyrosinase, Melan-A, MART-1, TRP-1/− 2
	Universal tumor antigens	Her2/neu, telomerase, survivin
	Oncoviral TAAs	HPV E6, E7, EBV-latent membrane proteins

**Fig. 1:**
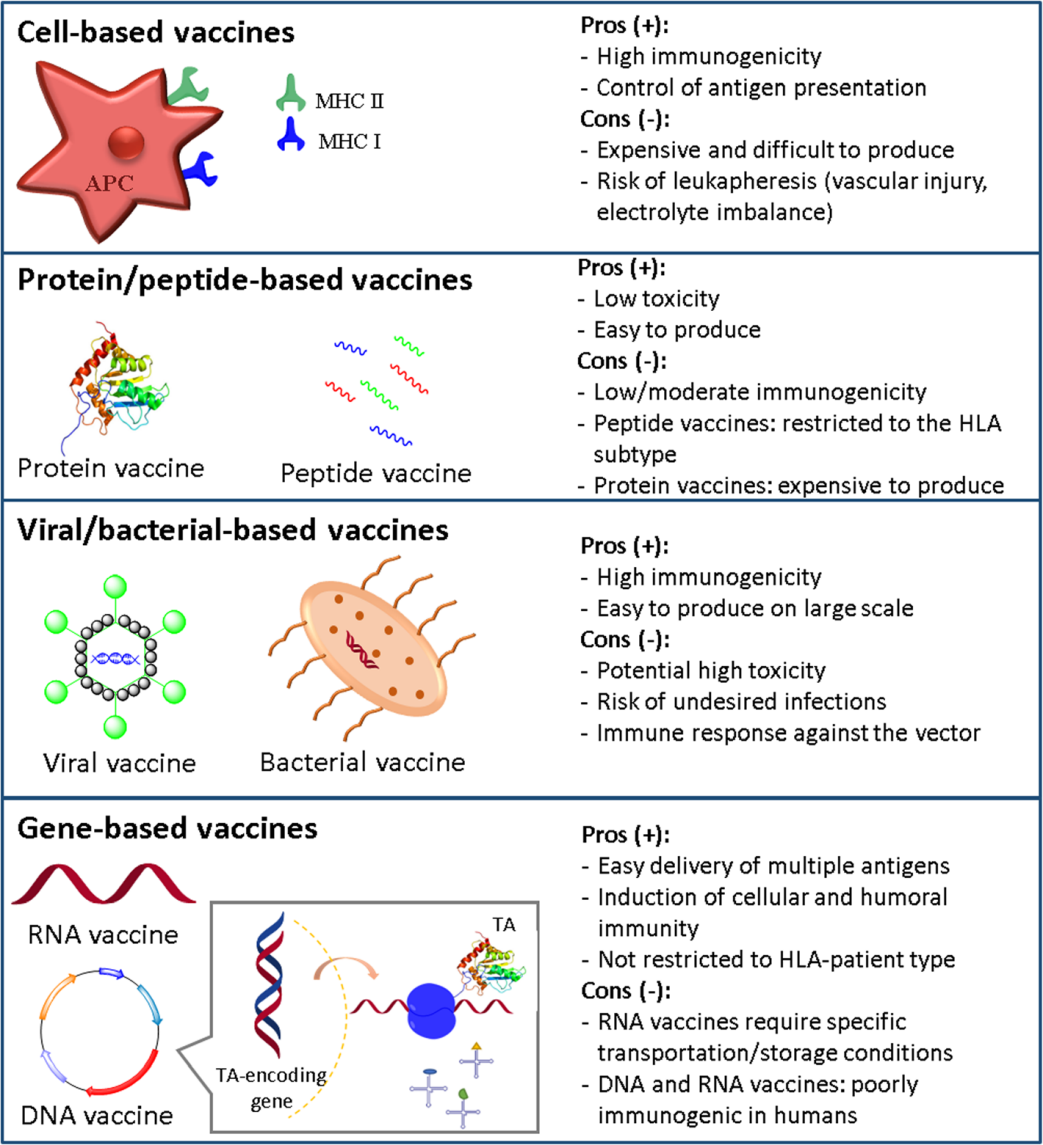
The different types of cancer vaccines

In the last few years, different types of cancer vaccines have been developed, i.e., formulations of TAs able to elicit an immune response to arrest the progression of cancer and prevent it from recurring [16]. These include cell-based vaccines, such as dendritic cell vaccines (e.g., Sipuleucel) [17] or whole tumor cells, protein/peptide vaccines [18], viral/bacterial-based vaccines [19, 20] and gene-based vaccines, including RNA and DNA vaccines [7, 21] (Fig. 1).

**Fig. 2:**
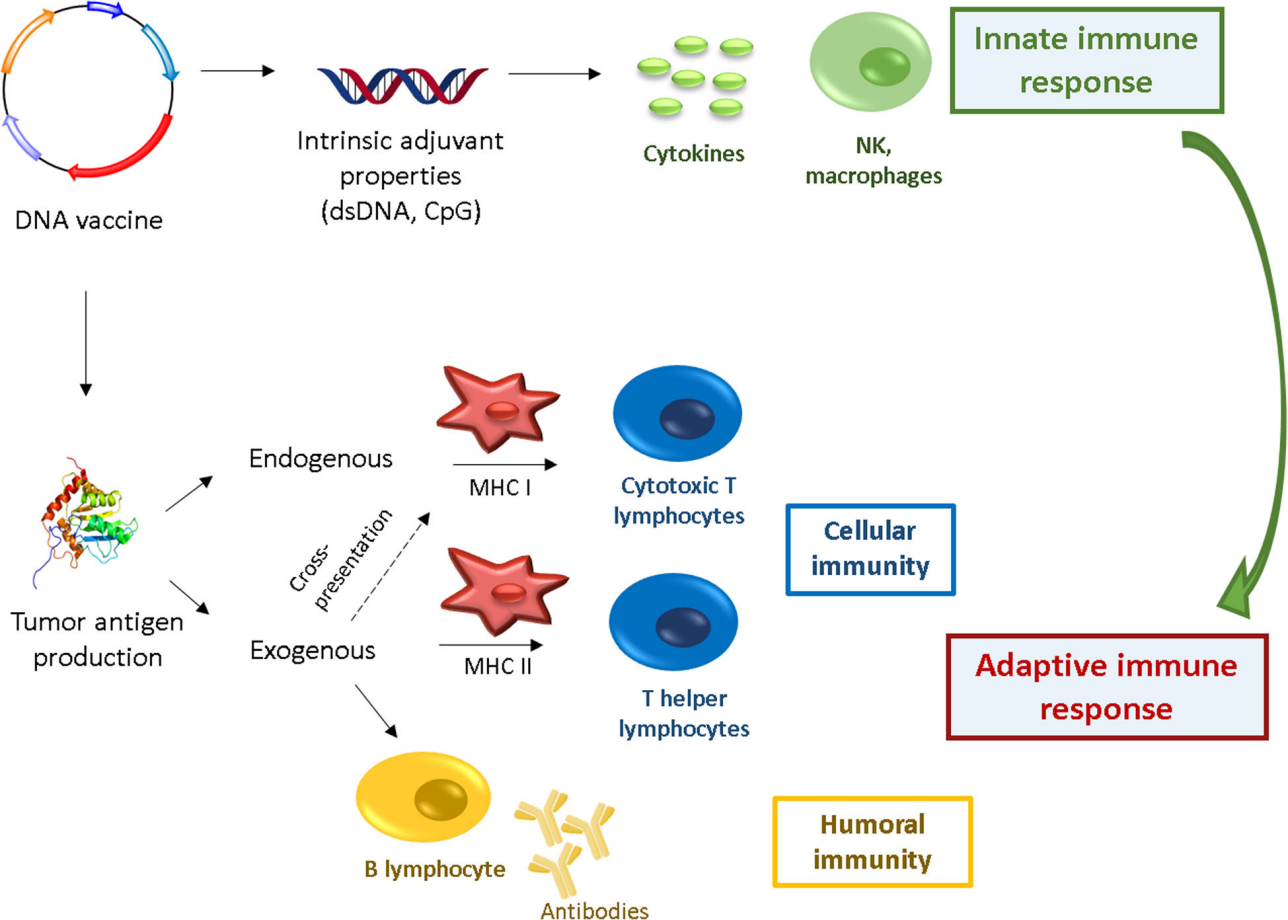
Innate and adaptive immune activation induced by DNA vaccines

In this context, DNA vaccination represents a promising strategy for harnessing the immune system. DNA vaccines are plasmids designed to deliver genes encoding TAs, eliciting or augmenting the adaptive immune response towards TA-bearing tumor cells. Cancer DNA vaccines can also induce the innate immune response, acting as powerful “danger signals” that stimulate several DNA-sensing pathways in the cytosol of transfected cells due to the presence of CpG motifs and the double stranded structure itself [22] (Fig. 2).

Until now, only one therapeutic cancer vaccine has been approved for human use (DC cancer vaccine, Sipuleucel T) [7], and most of the other cancer vaccines, including DNA vaccines, are still in clinical phase I or II. New developments in plasmid delivery and optimization and the combination with other therapies aim to improve the efficacy of DNA vaccines in preclinical and clinical studies to overcome their low immunogenicity in humans. In this review, we investigate the last 5-year preclinical studies and 10-year clinical studies using non-formulated (naked) DNA vaccines for cancer therapy. We also analyze the strategies that are being developed to overcome the actual limitations in cancer DNA vaccination to predict future trends in this field.

### Cancer DNA vaccines advantages and limitations

DNA-mediated immunization began in the 1990s, when a plasmid DNA encoding the influenza A nucleoprotein led to a protective and specific cytotoxic T lymphocyte (CTL) response [23]. Since then, DNA vaccines have been developed to treat a variety of pathologies, including allergies, infectious diseases, autoimmune diseases and cancer. DNA vaccines are based on bacterial plasmids that encode antigens and eventually encoded immunostimulatory molecules (IL-2, GM-CSF, etc.). They can be delivered by a variety of different routes, including intramuscular (IM), intradermal (ID), subcutaneous (SC) and mucosal. The most commonly used delivery strategies are physical methods, such as electroporation [24], sonoporation [25, 26], DNA tattooing [27], or gene gun [28], which are able to overcome the extra and intracellular barriers to transport DNA into the nucleus. Once in the nucleus, the antigen encoded by the DNA vaccine needs to be expressed and presented on major histocompatibility molecules (MHC) for T cell activation. An important advantage of DNA vaccines is that the encoded antigen can be presented by MHC class I and class II, thus activating both CD4 and CD8 T cells and, indirectly, humoral immunity [29]. Furthermore, the intrinsic elements of plasmid DNA can also activate the innate immune response due to the recognition of the double stranded DNA structure by cytosolic sensors [30, 31]. The induction of a protective and specific immune response has been demonstrated in mice against a variety of antigens, including hepatitis B surface and core antigens, HIV Env and Gag antigens, as well as TAs in different cancer models [32–35].

The antigen specificity and the safety of cancer DNA vaccines confer some advantages compared to other nonspecific and nontargeted therapies, which could have many side effects and cause extensive damage to normal tissues [9]. Cancer DNA vaccines promote a systemic immune response and thus are also effective on metastases, which are not easily removed by surgical intervention. In addition, unlike antibodies and small molecule inhibitors, DNA vaccines promote immunological memory [36, 37].

However, despite the improvement in the delivery techniques, DNA vaccines revealed poor immunogenicity in human trials [38, 39]. Some molecular approaches have been tested to improve their efficacy, such as codon optimization. This gene engineering technique permits the replacement of synonymous codons to increase protein production and plasmid immunogenicity [37]. Optimized cancer DNA vaccines demonstrated good efficacy in different preclinical models [37], especially in prophylactic models, and showed a great safety profile in humans. Unfortunately, the success in therapeutic vaccination is still limited even in preclinical models [37, 40]. This limitation is mostly due to the different mechanisms of resistance during tumor development, such as a loss or change of epitopes recognized by immune cells, T cell exhaustion, antigen tolerance, and the infiltration of immunosuppressive cells (regulatory T cells (Tregs), myeloid-derived suppressor cells (MDSCs), tumor-associated macrophages (TAMs), etc.), which produce immunosuppressive cytokines (TGF-β, IL-10, etc.) and a deprivation of nutrients and oxygen [41, 42]. Hence, new strategies are necessary to completely eradicate tumors.

The analysis of the last 5–10 years of preclinical and clinical trials revealed two main trends. First, a rational choice of the encoded antigen(s) can straighten the DNA vaccine immunogenicity and induce a broad immune response, overcoming the problems linked to antigen loss, modification and tolerance. Second, strategies that combine different therapies to prevent the infiltration of immunosuppressive cells and the production of immunosuppressive cytokines have been implemented to reduce immunosuppression in the tumor microenvironment (TME).

### Enhancement of DNA vaccine immunogenicity

#### Chimeric DNA vaccines

Chimeric DNA vaccines are vaccines that encode xenogeneic antigens. They are proteins or peptides derived from different species in which the sequence is significantly homologous with the self-ortholog [43]. The subtle differences between the epitopes of the orthologue and the native protein elicit T and B cell responses against the xenoantigen [13, 43]. Hence, xenogeneic antigens are recognized as “non-self-antigens”, thus circumventing immune tolerance while preserving an optimal homology to allow T cell recognition [13, 44]. During recent years, different studies have demonstrated the higher efficacy of xenogeneic antigens compared to autologous antigens [44, 45]. A complex DNA vaccine construct that delivers several xenogeneic epitopes dramatically increased the CTL antitumor activity [46]. The efficacy of DNA xenovaccines was also tested in dogs [47], leading to the approval of the first xenogeneic DNA vaccine against human tyrosinase, Oncept, for the treatment of oral malignant melanoma in dogs [43].

It is also possible to design hybrid plasmids, which code for chimeric proteins that include both xenogeneic and homologous antigen domains [13]. In this type of plasmid, the xenogeneic moiety can circumvent immune tolerance and induce a more potent cellular response, while the homologous sequence can stimulate the activation of a broader immune response [43]. Indeed, the chimeric protein produced by transfected cells can be taken up by DCs, thus activating the T cell immune response but it can also be recognized and internalized by B cells [43]. Quaglino et al. found that the plasmid encoding the chimeric neu-Her-2 antigen was superior to both the fully autologous and the fully xenogeneic vaccines in inducing a protective antitumor immune response against ErbB2^+^ tumors [48]. Starting from these results, other DNA vaccines were constructed by shuffling genes from mouse, rat, human and other species, improving the antigen immunogenicity and vaccine efficacy [49–52]. DNA xenovaccination has also been tested in the clinic in melanoma patients, with encouraging results [53, 54], and one clinical study (NCT00096629) using the human and murine prostate-specific membrane antigen is ongoing (Table 2).

**Table 2:** Clinical trials in cancer DNA vaccination (2009–2019)

Cancer type	Phase	Study start	DNA vaccine/encoded antigen	Combination therapy	Treatment schedule	DNA delivery	References
Breast cancer	I	2015	Personalized polyepitope	/	- Vaccine: 4 mg, at D 1, 29 and 57	IM EP	NCT02348320
	I	2015	Mammaglobin-A antigen	Anastrozole, Letrozole, Tamoxifen, Exemestane, Goserelin, endocrine therapy	- Vaccine: 2 injections in the deltoid or lateralis muscles, at D 28, 56 and 84 - Endocrine therapy: to be determined by the treating physicians	IM EP	NCT02204098
	I	2015	pUMVC3-CD105/Yb-1/SOX2/CDH3/MDM2-polyepitope: mammalian expression vector pUMVC3 + CD105, Y-box binding protein-1, SRY-box 2, cadherin 3, murine double minute 2	rhuGM-CSF, adjuvant therapy	- Vaccine: every 28 D for 3 M, then an injection at 6 and 12 M - rhGM-CSF: ID every 28 D for 3 M	ID	NCT02157051
	I	2016	pUMVC3-IGFBP2-HER2-IGF1R: pUMVC3 vector + insulin-like growth factor binding protein-2 (IGFBP2), HER2 and insulin-like growth factor 1 receptor precursor (IGF-1R)	GM-CSF (Sargramostim), adjuvant therapy	Vaccine: D 1 and every 28 D, 3 times - GM-CSF: the same as the vaccine	ID	NCT02780401
	I	2018	Neoantigens	Durvalumab (anti-PD-L1 antibody), immune therapy	- Vaccine: 2 injections in 2 different sites, 3 M after the standard of care (D 1) and then D 29, 57, 85, 113 and 141 - Durvalumab: 1500 mg every 4 W, at D 85	EP	NCT03199040
Prostate cancer	II	2009	pTGV, encoding prostatic acid phosphatase (PAP)	rhGM-CSF, adjuvant therapy	- Vaccine: 100 μg, every 2 W for the first 12 W and then every 12 W, according to the immune response - rhGM-CSF: 200 μg every 3 M	ID	NCT00849121
	II	2011	pTGV-HP	rhGM-CSF, adjuvant therapy	- Vaccine: 100 μg, every 2 W for 6 times and then every 3 M for 2 years - rhGM-CSF: the same as the vaccine, but 208 μg of dose	ID	NCT01341652
	II	2013	pTGV-HP	Sipuleucel-T, autologous peripheral blood mononuclear cells with antigen presenting dendritic cells that have been activated ex vivo with a recombinant fusion protein (PA2024) consisting of PAP linked to GM-CSF, immune therapy	- Vaccine: at W 6, 8, 10, 12, M 6 and 12 - Sipuleucel-T: W 0, 2 and 4	/	NCT01706458
	I	2015	pTGV-AR	GM-CSF, adjuvant therapy	- Vaccine: 100 μg, 6 doses every 2 W and then 12, 24, 36 and 48 or 100 μg at W 0, 2, 12, 14, 24, 26, 36, 38, 48, 50 - GM-CSF: 200 μg co-injected with the vaccine	/	NCT02411786
	I/II	2015	pTGV-HP	Pembrolizumab (anti-PD1 antibody), immune therapy; rhGM-CSF, adjuvant therapy	- Vaccine: 100 μg, at D 1, 15, 29, 43, 57 and 71 - Pembrilizumab: 2 mg/kg, IV, every 3 W, at D 1, 22, 43, 64 or D 85, 106, 127 and 148 - rhGM-CSF: 208 μg, ID, every 3 W	ID	NCT02499835
	II	2018	pTGV-HP	Nivolumab, GM-CSF	- Nivolumab: 240 mg IV every 2 W × 6 beginning D 1, then every 4 W × 9 beginning W 12) - rhGM-CSF: 208 μg, ID every 2 W × 4 beginning W 4, then every 4 W × 9 beginning W 12 - pTVG-HP: 100 μg, every 2 W × 6, beginning at D 1; then every 4 W × 9, beginning at W 12	ID	NCT03600350
	I	2018	Neoantigens	Nivolumab/Ipilimumab and Prostvac	- Priming with Prostavac (1 mg/kg, every 3 W, 2 doses) and the ICB (3 mg/kg, every 3 W, 6 doses) - Vaccine: 4 mg at 2 different sites, 6 times every 28 D	IM EP	NCT03532217
Cervical cancer	I/II	2015	VB10.16: composed by E6/E7 antigen of HPV16 + dimerization entity + APC binding protein	/	- Vaccine: 3 mg, W 0, 3, 6 or W 0, 4, 12	Lateral deltoid mucle	NCT02529930
	II	2015	GX-188E, encoding E6/E7 fusion protein of HPV 16 and 18, plus the immune-enhancer, Fms-like tyrosine kinase-3 ligand (FLT3L)	/	- Vaccine: 1 mg, W 0, 4 and 12	IM	NCT02596243
	I/II	2017	MEDI0457 = INO3112 = VGX-3100 (, encoding E6 and E7 proteins of HPV types 16 and 18) + INO-9012 (hIL-12)	Darvalumab (anti PD-L1 antibody), immune therapy	- Vaccine: / - Darvalumab: 1500 mg, IV, every 4 W	IM EP	NCT03162224
	/	2017	GX-188E	GX-I7 encoding IL7 receptor agonist, Imiquimod, adjuvant therapy	- Vaccine: 1 mg, 3 times - GX-I7: 3 mg, locally on the cervix, 4 times - Imiquimod: 12.5 mg, administered locally on the cervix, 8 times	IM	NCT03206138
	III	2017	VGX-3100	/	- Vaccine: 1 ml on D 0, W 4 and W 12	IM EP	NCT03185013
	I/II	2018	GX-188E	Pembrolizumab	- Vaccine: 1.0 mg/0.5 ml - Pembrolizumab: 100 mg/4 mL, IV	IM EP	NCT03444376
	II	2018	VGX-3100	/	- Vaccine: over 10 s for 4 doses in W 0, 4, 12, and 24	IM EP	NCT03603808
	II	2018	VGX-3100	Darvalumab	- Vaccine: W 1, 3, 7, and 12 - Darvalumab: W 4, 8, and 12	IM EP	NCT03439085
	III	2019	VGX-3100	/	- Vaccine: 1 ml on D 0, W 4 and W 12	IM EP	NCT03721978
Ovarian cancer	I	2012	pUMVC3-hIGFBP2 multiepitope: mammalian vector pUMVC3 + human IGFBP2	/	- Vaccine: monthly, for 3 M	ID	NCT01322802
	II	2017	pUMVC3-hIGFBP2 multiepitope	Carboplatin, Paclitaxel, chemotherapy	- Vaccine: 2 W after the chemotherapy and every 3 W - Chemotherapy: IV, 2 W before the vaccine	ID	NCT03029611
Pancreatic cancer	I	2018	Personalized neoantigen: pING vector + prioritized neoantigens + mesothelin epitopes	Chemotherapy	- Vaccine: W 1, 5, 9, 13, 17 and 21 - Chemotherapy: at W 1, 5, 9, 13, 17, 21, 25 and 77, after surgery and before vaccination	IM EP	NCT03122106
Glioblastoma	I/II	2018	INO-5401 (3 separate DNA plasmids targeting Wilms tumor gene-1 (WT1) antigen, prostate-specific membrane antigen (PSMA) and human telomerase reverse transcriptase (hTERT) genes)	Cemiplimab, radiation and chemotherapy; INO-9012	- Vaccine: 3 mg at D 0, every 3 W × 4 doses, and then every 9 W - INO-9012: 1 mg at the same time of the vaccine - Cemiplimab: IV, every 3 W at a dose of 350 mg per dose - Radiation therapy: 42 days after surgical intervention, and should start approximately 2 W after D 0 - Temozolomide: daily during radiation therapy, at a dose of 75 mg/m^2^	IM EP	NCT03491683
Melanoma	Early I	2018	IFx-Hu2.0 coding for Emm55 Streptococcal Antigen	/	- Vaccine: 100 μg in 200 μL per lesion	Intralesion	NCT03655756
Renal cell carcinoma	II	2019	Neoantigens	Darvalumab Tremelimumab	- Durvalumab: IV at a dose of 10 mg/kg over the course of 60 min, every 2 W, 8 doses - Tremelimumab: at a dose of 1 mg/kg over the course of 60 min, every 4 W, 4 cycles - Vaccine: D 1 of the first 28 D cycle of treatment with durvalumab and tremelimumab, every 2 W	IM EP	NCT03598816
Solid tumors	I	2014	hTERT	/	- Vaccine: 100, 400 and 800 μg as a single agent, every 4 W × 3 cycles	ID EP	NCT02301754
Anal neoplasm	II	2018	VGX-3100	/	- Vaccine: 1 ml on D 0, W 4 and W 12, and potentially W 40	IM EP	NCT03499795
Urothelial carcinoma	I/II	2018	INO-5401	INO-9012, Atezolizumab	- Vaccine: 9 mg, every 3 W × 4 doses then every 6 W × 6 additional doses, thereafter every 12 W - INO-9012: administered with the vaccine - Atezolizumab: IV infusion every 3 W	IM EP	NCT03502785

M = month; W = week; D = day; EP = electroporation; IM = intramuscular; ID = intradermal; IV = intravenous

#### Neoantigen DNA vaccines and personalized vaccination

Most anticancer DNA vaccines, both past and present, immunize using nonmutated TAs. However, these antigens are often present in normal or germline tissues, which can prevent a strong immune activation because of immune tolerance [55]. Several clinical trials using nonmutated TAs have failed to demonstrate beneficial effects compared with the standard of care treatment [14]. In contrast, neoantigens are the result of tumor-specific DNA alterations that create new epitopes. Due to their specific expression in cancer tissue and the potential lack of side effects, they represent ideal targets against cancer and can be used in the design of cancer vaccines [56, 57]. They can also turn “cold” tumors into “hot” ones and mediate the upregulation of PD-L1 in the TME, thus extending the applicability of the anti-PD-1/PD-L1 immunotherapy [58]. Neoantigens are presented by APCs to CD4+ and CD8+ T cells to activate an immune response. They are highly tumor-specific and, therefore, they represent an attractive immunotherapy target. It is expected that they are not affected by T cell tolerance, as they may be recognized as non-self by the host immune system and, thus, generate a specific anti-tumor response [59, 60]. Their identification starts with exon sequencing from a tumor biopsy. Then, mutations are identified compared to whole exome data from normal tissue. Prediction algorithms select those antigens that are recognized by MHC class I or II. Finally, in vitro and in vivo studies validate their ability to stimulate the CD8+ immune response, especially a CD4 response [61, 62]. However, not all peptides are immunogenic, and identifying which mutations are targeted by the immune system is currently a subject of great interest. Hence, the prediction of the immune response to neoantigens needs to be optimized. Assessing the immunogenicity of each neoepitope is not reasonably applicable on a large scale. Current computational approaches are being refined to improve the accuracy of neoantigen identification and are discussed in detail in [63]. Integrated pipelines will need to be developed beginning with tumor genomic characterization, variant analysis, and the accurate prediction of which mutations are likely to give rise to tumor-specific neoantigens [64]. Other hurdles are associated with the use of personalized neoantigens for cancer immunotherapy, such as the manufacturing time. The median period for the discovery and production of a personalized vaccine is approximately 4.5 months [65]. In particular, the time from the selection of mutations to vaccine release ranges from approximately 89–160 days [66]. This amount of time has to be reduced to cure patients with metastatic disease. Another issue concerns the genetic heterogeneity of tumors [67]. Thus, targeting a unique neoantigen would probably lead to the selection of antigen non-expressing tumor cells. It has been demonstrated that the use of a poly-epitope neoantigen RNA vaccine encoding up to 10 neoantigens was effective in 8/13 melanoma patients who were completely tumor-free after one year [66]. Compared to RNA and peptide vaccines, DNA vaccines seem to elicit a more potent CD8 response against the encoded neoantigens, making them more attractive for cancer vaccination [60, 68]. Hence, once identified, the neoantigen can be cloned into a DNA vaccine. This personalization permits the design of cancer vaccines tailored to each patient.

#### Polyepitope DNA vaccines

An advantage of DNA vaccines is the possibility of delivering several antigen genes in the same construct, at the same time and with the same delivery method. The presence of immunodominant and unconventional epitopes simultaneously delivered by a polyepitope DNA vaccine can induce a broad CTL response specific to multiple antigens [69]. In this way, it is possible to overcome the antigen mutation or deletion by tumor cells, the variation or absence of the appropriate T cell repertoire and the MHC haplotype in patients [69].

When designing a poly-epitope DNA vaccine, many parameters should be considered. First, the competition for antigen recognition at the surface of the APC and the affinity of the selected epitopes for MHC molecules should be considered [70, 71]. Palmowski et al. demonstrated that the use of an MHC class I polyepitope vaccine leads to the preferential expansion of CTLs with a single immunodominant specificity [72, 73]. In addition, the affinity of the selected epitopes for MHC molecules and transporters could influence the CTL immunodominance and the consequent immune response [70].

Second, although the CD8 T cell response has been considered to be the main protagonist in the antitumor immune response resulting from vaccination, the insertion of an epitope/antigen recognized by CD4 T cells into a DNA vaccine could activate a broader and stronger immune response. Several studies suggest the importance of the CD4 T cell population for cancer immunotherapy [74, 75]. Recently, it has been demonstrated that CD4 T cells recognize a higher number of neoantigens than previously known and can generate potent antitumor responses [62, 76]. Hence, a coordinated CD4 and CD8 response is necessary for the complete eradication of a tumor [76]. T helper (Th) peptides have already been used in combination with DNA vaccines to increase the activation of Th cells, thus further eliciting the CTL immune response [77–82]. An example of a Th epitope is the pan DR *epitope (*PADRE). This synthetic Th epitope, encoded in a DNA vaccine and administered with an antigen-encoding plasmid, increased the number of antigen-specific CD8 T cells, resulting in potent protective and therapeutic antitumor effects [83]. Other studies demonstrated that a PADRE-encoding DNA generated CD4 Th1 cells that play an important role in maintaining long-term memory responses, helping the activity of CD8 T cells [84].

Many techniques have been developed to find new epitopes. These studies led to the identification of NY-ESO-1, MelanA/MART-1, SSX4, MELOE-1 and TRAG-3 in melanoma, EphA2 and MAGE-6 in renal cell carcinoma, CEA, MAGE-3 and telomerase in lung carcinoma, TRAG-3 in breast carcinoma, and NY-ESO-1, p53 and SSX4 in ovarian cancer, among others [85]. Some of these tumor antigens recognized by CD4 T cells belong to the same categories as those recognized by cytotoxic CD8 T cells [75].

Finally, it is important to identify the most immunogenic epitopes derived from tumor antigens. New in silico techniques are being developed to improve the prediction of epitope immunogenicity to design a poly-epitope vaccine. They not only consider the binding affinity to the MHC and the different HLA subtypes but also the conformation and interaction with the HLA, immunodominance vs tolerance, etc. [86]

Many recent preclinical studies have investigated the use of polyepitope DNA vaccines to reach a broad immune response. As a result, an increased IFNg production, a higher Th and CTL response [86, 87], and a general decrease in the tumor growth rate and metastasis formation were observed in different types of cancer models [88, 89]. Some preclinical studies focus on the HPV model, using DNA vaccines encoding E6 and E7 molecules [90], or E7 with a helper epitope [88]. Another example is SCT-KDR2, which encodes the mouse β2microglobulin + KDR2 (VEGFR2 antigen peptide) + MHC class I H-2D^b^, in a B16 melanoma tumor model [89]. A non-exhaustive list of the most recent preclinical trials (in the last 5 years) can be found in Table 3. Additionally, many clinical trials are testing the safety and efficacy of polyepitope DNA vaccines, such as NCT02348320 and NCT02157051 for breast cancer, NCT02172911 for cervical cancer, and NCT01322802 and NCT03029611 for ovarian cancer. In particular, in the clinical studies NCT02348320 and NCT03199040, a personalized polyepitope vaccine against breast cancer is being used, as well as in the NCT03122106 for pancreatic cancer, and the results will help to establish the relevance of this vaccine strategy. This would address tumor heterogeneity and the loss of immunogenicity associated with TAAs, which accounts for the failure of the current anticancer treatments [58]. A complete list of the ongoing clinical trials could be found in Table 2.

**Table 3:** Preclinical studies in cancer DNA vaccination (2015–2018)

Cancer type	Animal	DNA vaccine	Combination therapies	Protocol	DNA vaccine delivery	Results	Year, ref.
Cervical cancer (TC-1 cells)	C57BL/6 mice	HPV plasmid encoding E6 and E7 antigens	pVAX1-ISG15 encoding an optimized mouse adjuvant ISG15	Therapeutic vaccination Tumor: 5 × 10^4^ TC-1 tumor cells, SC Vaccine and adjuvant: 7 D after tumor implantation, followed by 3 boosts weekly	IM EP, in the tibialis anterior muscle	- Strong HPV E7-specific CD8+ T cell immune response - Increase in INF-γ secretion - 6/10 mice were tumor-free at D 42	2015 [90]
Cervical cancer (TC-1 cells)	C57BL/6 mice	pcDNA3.1-E7, encoding E7 antigen of HPV16	Monophosphoryl lipid A (MPL, TLR4 agonist) and α-galactosylceramide (GalCer)	Therapeutic vaccination Tumor: 2 × 10^5^ TC-1 tumor cells, SC Vaccine: 100 μg, 7 D after tumor implantation, followed by 2 boosts weekly Adjuvant: 25 μg of MPL and 1 μg of GalCer, SC with the vaccine	SC	- CTL-specific cytolytic activity - Higher INF-γ, IL-4 and IL-12 production - Decrease in tumor growth if both adjuvants were administered	2016 [136]
Cervical cancer (TC-1 cells)	C57BL/6 mice	HELP-E7SH, encoding E7 antigen of HPV16 and a helper epitope to stimulate CD4^+^ response	Abs against CD70, CTLA-4, PD-1; agonistic Ab to CD27	Therapeutic vaccination Tumor: 1 × 10^5^ TC-1 tumor cells, at D 0 before the vaccination, SC Vaccine: 15 μl of a 2 mg/ml DNA solution, on D 0, 3 and 6 Abs: 100 μg, at D 0, 3 and 6 (CD70 also at D 9), IP	Tattoo (intraepidermal vaccination)	- Help epitopes increased E7-specific CD8^+^ response in lymph nodes and spleen - CTLA4 and PD-1 did not promote CTL priming, if not combined with CD27 or the vaccine - CD27 agonism + anti-PD1 improved mice survival, albeit CD27 + anti-CTLA4 further increased the CTL response	2016 [35]
Cervical cancer (TC-1 cells)	C57BL/6 mice	pVAX1-gDE7, encoding HPV-16 E7 protein fused to HSV-1 gD protein	pcDNA3-IL2 encoding murine IL-2; anti-Gr1 Ab	Therapeutic vaccination Tumor: 7.5 × 10^4^ TC-1 tumor cells, at D 0 before the vaccination, SC Vaccine and adjuvant: 50 μg alone or in combination with 50 μg of the adjuvant, at D 3, 2 doses, weekly Anti-Gr1: 200 μg, once/W for 6 IP injections, at D 7–10, 5 doses, weekly	IM	- Vaccination with the 2 plasmids avoided MDSC accumulation - Combination of the vaccines and anti-Gr1 antibody increased mice survival, completely eradicating the tumor	2016 [95]
Cervical cancer (TC-1 cells)	C57BL/6 mice	pcDNA3.1-E7, encoding HPV-16 E7 antigen	melatonin	Therapeutic vaccination Tumor: 2 × 10^5^ TC-1 cells at D0, before the vaccination, SC Vaccine: 90 μg, 3 times, at 7 D interval Melatonin: 50 or 100 mg/kg	SC	- Production of HPV16 E7-specific CTL - Increase of IFNg and TNFa in the TME - Tumor volume reduction	2018 [34]
Cervical cancer (TC-1 cells)	C57BL/6 mice	dbDNA, encoding HPV16 E6 and E7	/	Therapeutic vaccination Tumor: 5 × 10^4^ TC-1 cells, SC, at D0 Vaccine: at D3, 25 μg/hind, boost after 7 W	IM in the anterior tibialis and IM EP in the quadriceps	- Delay in the tumor growth - High levels of IFNg-secreting Th cells - Production of IgG1 - IL-12 production and low IL10	2018 [137]
Cervical cancer (HPV)	BALB/c mice	pNGVL4a-hCRTE6E7L2, expressing the HPV16 E6, E7 and L2 antigens	/	Prophylactic vaccination Vaccine: 3 injections, biweekly Tumor: 12 μl of HPV16 PsV, 19 D after the last vaccination	IM EP	- Production of Ab against E6, E7 and L2; - Protection of 3/5 of mice from the challenge, but without a significant difference compared to the control group	2017 [138]
Lobular carcinoma (TUBO cells)	BALB/c mice	pAmot, coding human p80 Amot (Angiomotin), antiangiogenic	/	Therapeutic vaccination Tumor: 10^5^ TUBO cells, SC Vaccine: 50 μg, at D 7	IM EP in the quadriceps muscle	- Delay in tumor progression - Heterogeneous changes in the tumor region following antiangiogenetic treatment	2015 [139]
Murine breast cancer (D2F2 cells)	BALB/c mice	pVAX-E2A, encoding Her2/neu antigen	pVAX-CCL4, encoding CCL4, chemoattractant for immune effector cells	Prophylactic vaccination Vaccine: 2 × 100 μg, on D 1 and 15 Tumor: 2 × 10^5^ Her2/neu^+^ cells, on D 25, SC	IM	- With the combined therapy, 26% of mice remained tumor-free (CCL4 improved tumor protection) - CCL4 produced a Th1 anti-Her2/neu response	2016 [140]
Murine breast cancer (4 T1 cells)	BALB/c mice	CpVR-FAP, encoding fibroblast associated protein (FAP)	Cyclophosphamide, chemotherapy agent	Therapeutic vaccination Tumor: 2 × 10^4^ 4 T1 cells, at D 0, SC Vaccine: 100 μg, on D 2, 9 and 16 after tumor injection Cyclophosphamide: 50 mg/kg, on D 1, 8, 15, IP	IM in the tibialis anterior muscle	- Combination therapy increased median survival time of mice - Suppression of IL-10, VEGFα and CXCL12 mRNA expression	2016 [115]
Murine breast cancer (4 T1 cells)	BALB/c mice	CpVR-FAP, encoding FAP	/	Prophylactic and therapeutic vaccination Tumor: 2 × 10^4^ 4 T1 cells, SC Vaccine: 100 μg, on D 2, 9 and 16 after tumor injection or 3 times every 2 W before tumor injection	IM in the tibialis anterior muscle	- Specific CTL response against FAP - Increased IL-2 production - Delay of the tumor growth also in therapeutic setting - Decrease in FAP expression without impairing wound healing	2016 [141]
Murine breast cancer (4 T1 cells)	BALB/c mice	pVAX1-mCr-1, encoding mouse Cripto-1 oncofetal protein	/	Prophylactic vaccination Vaccine: 40 μg Tumor: 2 × 10^5^ 4 T1 mCr-1 cells, W12	ID EP	- Humoral response against Cr-1 - Protective immune response against cancer stem cells - Reduced lung metastasis	2018 [142]
Colon cancer (colon 26/β-gal cells)	BALB/c mice	pcDNA3/β-gal encoding β-galactosidase	pCAGGS/FasL encoding Fas ligand	Prophylactic vaccination Vaccine and adjuvant: 50 μg + 1 μM cardiotoxin to facilitate DNA uptake Tumor: 10^6^ Colon 26/β-gal cells, 21 D after vaccine injection, SC	/	- The combined therapy decreased tumor growth rate - Production of Abs anti-β-gal	2015 [143]
Colon cancer (CT26/HER2 cells)	BALB/c mice	pVAX1-HER2, coding HER2 antigen	Gemcitabine, chemotherapy agent; anti-Gr1 antibody; anti-PD-L1 Ab	Prophylactic and therapeutic vaccination Tumor: 3–5 × 10^5^ CT26/HER2 cells, SC Vaccine: 50 μg Anti-PD-L1 and Gr-1 Ab: 200 μg and 250 μg, respectively, IP Gemcitabine: 75 μg/g, 2 times/W, IP	IM EP	- In prophylactic vaccination, the combination of vaccine + anti-PD-L1 Ab failed to delay tumor growth - The addition of anti-Gr1 or gemcitabine delayed tumor growth	2017 [144]
Colon cancer (CT26 cells)	Balb/c mice	CpVR-MS and CpDV-IL2-MS, encoding a fusion gene of human surviving S8 and human 33 MUC1, plus IL2)	Ad-MS (Adenovirus)	Therapeutic vaccination Tumor: 10^6^ CT26 cells, SC Vaccine: 100 μg, twice Ad-MS: 10^8^ pfu, D1, 15 and 29	IM	- Specific immune response in splenocytes - Upregulation of CCL-19 and GM-CSF - Downregulation of PD-L1 and MMP-9	2018 [145]
Colorectal cancer (CT- 26/NIS cells)	BALB/c mice	pcDNA-hNIS, expressing human sodium/io- dide symporter (hNIS)	/	Vaccine: 100 μg, 3 times at 2 W intervals Tumor: 2 W after the final hNIS DNA injection, 5 × 10^5^ (left) or 1 × 10^5^ (right) CT-26/NIS cells, SC	ID	- Increase of IgG2a/IgG1 ratio - Increase of INF-g secreting cells and IFN-g production - Th1 response - Slower tumor growth	2018 [146]
Melanoma (B16F10-β-hCG cells)	C57BL/6 mice	CAVE = pSVK-VEGFR2-GFc-IL12, Semliki Forest Virus expressing VEGFR2 and IL-12	CAVA = SFV replicon DNA vaccine targeting surviving and hCG antigens	Prophylactic vaccination Vaccine: 10 μg of CAVA/CAVE, 3 times at 10 D of interval Tumor: 7.5 × 10^4^ B16F10-β-hCG cells, 7 D after the last immunization, SC	IM EP	- Combination of the 2 vaccines delayed tumor growth more efficiently than the single vaccine and increased mice survival - CAVE + CAVA decreased microvessel density	2015 [147]
Melanoma (B16F10 cells)	C57BL/6 mice	pSPD-gp100-CD40L, encoding gp100 inserted between mouse Surfactant Protein D (SPD) and CD40L	pIL-12, encoding IL-12p70; pcDNA3.1-GM-CSF encoding GM-CSF	Therapeutic vaccination Tumor: 5 × 10^4^ B16F10 cells, ID Vaccine and adjuvants: 80 μg vaccine + 20 μg of each adjuvant plasmid, on D 3, 10 and 17	IM in hind quadriceps muscles	- Vaccine alone did not delay tumor growth, but the combination with the 2 adjuvants was very effective in increasing mice survival	2015 [94]
Melanoma (B16F10 cells)	C57BL/6 mice	pVAX1-MUCI, encoding mucin I glycoprotein	pVAX1-Flt3L, encoding Fms-like tyrosinase 3-ligand	Therapeutic vaccination Tumor: 1 × 10^6^ B16F10 cells, SC Vaccine + adjuvant: 50 μg, priming when tumors were palpable, boosts after 7 and 14 D	IM EP	- Specific CTL and antibodies - Tumor growth suppression	2018 [148]
Melanoma (B16 cells)	C57BL/6 mice	p-mBAP31 and p-LAMP/mBAP31 = p43 and p43- Lysosomal Associated Membrane Protein (LAMP) vectors + mouse B-cell receptor-associated protein (mBAP)	/	Therapeutic vaccination Tumor: 5 × 10^4^ B16 cells, at D 0, SC Vaccine: 50 μg, at D 3, 10, 17 and 24	SC	- No evidence of autoimmune disorders - High INF-γ production, especially using LAMP vaccine - LAMP vaccine increased the CTL cytotoxicity - Suppression of tumor growth, especially using LAMP vaccine	2015 [149]
Melanoma	Horses	Minimalistic immunogenically defined gene expression (MIDGE)-Th1 vector + eqIL12 and IL-1beta receptor antagonist protein (ILRAP)-eqIL18	hgp100MIDGE-Th1; htyrMIDGE-Th1	Therapeutic vaccination Tumor: horses were already affected by melanoma Vaccine and adjuvants: 500 μg ID peritumorally and 500 μg IM into the semimembranosus muscle, 3 times	ID and IM	- Vaccine was safe and well-tolerated, except an increase in the body temperature on the day after injection and signs of acute inflammation - Tumor volume was reduced by 28.5%, but without significant differences if adjuvants were added	2015 [150]
Melanoma (B16F10-OVA cells)	C57BL/6 mice	pVAX2-OVA, encoding ovalbumin; pVAX2-gp100, encoding gp100	pVAX2-HIV-1 Gag	Prophylactic and therapeutic vaccination Vaccine: 1 μg (p-OVA) or 50 μg (p-gp100), at D 2, 9 and 16 (therapeutic) or 3 times every 2 W before the tumor challenge (prophylactic) Adjuvant: 1 μg, co-administered with the vaccine Tumor: 1 × 10^5^ B16F10-OVA cells at D 0 (therapeutic) or 2 W after the last vaccine injection (prophylactic)	IM EP	- Delay of tumor growth and increase in mice survival - Codelivery of the adjuvant-encoding plasmid polarized the immune response towards a Th1-like phenotype	2016 [40]
Mastocytoma (P815 cells)	DBA/2 mice	Differently optimized pVAX2-P1A vaccines, encoding P815A	/	Prophylactic and therapeutic vaccination Vaccine: 50 μg, at D 2, 9 and 16 (therapeutic) or 3 times every 2 W before the tumor challenge (prophylactic) Tumor: 1 × 10^6^ P815 cells at D 0 (therapeutic) or 2 W after the last vaccine injection (prophylactic)	IM EP	- Delay of tumor growth and increase in mice survival Activation of innate immunity related to the different CpG motif amount inside the P1A gene	2017 [37]
Mastocytoma (P815 cells)	DBA/2 mice	Optimized pVAX2-P1A vaccine, encoding P815A	Anti-CTLA4, anti-PD1	Therapeutic vaccination Tumor: 1 × 10^6^ P815 cells at D 0 Vaccine: 50 μg, at D 2, 9 and 16 Anti-CTLA4, anti-PD1: 100 μg at D 3, 6 and 9	IM EP	- Survival reached 90% - Increase of specific T cell infiltration in the TME - Increase of IL-12 production - Decrease of metastasis formation	2018 [109]
Malignant tumor	HLA-A2.1/Kb transgenic mice	p-GST-YL66, against multiepitope YL66 (from COX2 and MAGE4), linked with membrane permeable Tat-PTD and the universal Th epitope	/	Not available	/	- CTL-mediated tumor cell lysis in vitro and in vivo	2017 [87]
Colorectal cancer (MC32 cells)	C57BL/6 mice	Pc-DNA3-CEA, carcinoembryonic antigen (CEA)	Ab 4-1BB	Prophylactic and therapeutic vaccination Tumor: 1–5 × 10^5^ MC32 cells, SC Vaccine: 50 μg, at 1 W of interval Ab anti-4-1BB: 50 μg, systemically, after vaccine injection	IM EP	- Antigen-specific CTL activity and tumor-protective immune response in prophylactic model - Ab 4-1BB increased CTL lytic activity - MC32 cells resisted to CEA DNA vaccination by loss of antigen presentation to CEA-specific CTL in therapeutic model	2015 [151]
Melanoma (B16), carcinoma (3LL)	C57BL/6 mice	SCT-KDR2, encoding the mouse β2microglobulin + KDR2 (VEGFR2 antigen peptide) + MHC class I H-2D^b^, subcloned into pdDNA3.1	/	Prophylactic vaccination Vaccine: 50 μg, 3 times, at 1 W of interval Tumor: 10^5^ B16 cells or 2 × 10^5^ 3LL cells, 10 D after the last vaccination, SC	ID	- CTL response to VEGFR2 - Inhibition of tumor-induced angiogenesis - Inhibition of tumor metastasis	2015 [89]
Sarcoma	HHDII-DR1 mice	SSX2-optimized vaccine, encoding modified cancer testis antigen	Ab anti-PD-1/L1	Prophylactic and therapeutic vaccination Vaccine: 100 μg, 6 times, every 2 W (prophylactic) or weekly the day after the tumor injection (therapeutic) Tumor: 2 × 10^4^ SSX2-expressing sarcoma cells An anti-PD1/L1: 100 μg, IP on the day following each vaccination	ID	- Optimized vaccine elicited inferior antitumor effect relative to the native vaccine - Increase of PD-L1 expression on tumor cells - CTL from immunized mice expressed more PD-1, increasing the antitumor efficacy of the combination with ICB	2015 [152]
Kidney cancer (RenCa cells)	BALB/c mice	pVAX1-G250-F2A-CTLA4, containing the co-expression gene G250-CTLA4, linked by Furin-2A (F2A)	/	Therapeutic vaccination Tumor: 10^5^ RenCa cells, SC Vaccine: 50 μg, at D 7, 17 and 27	EP	- Humoral and cellular-specific immune response against CTLA4 and G250 - Increase in INFγ and IL-4 (Th1/2 response) - Tumor growth rate decreased	2017 [153]

Keywords search “cancer plasmid DNA vaccine”, from 2015 to 2018. D = day, W = week, M = month

A good option to further optimize the efficacy of cancer DNA vaccination could be the combination of the 3 cited approaches, designing a poly-epitope chimeric vaccine containing specific neoantigens. In the clinic, this could reduce the number of nonresponding patients by developing a stronger and more complete immune response.

### Combination of DNA vaccines with other therapies

In the analyzed preclinical (Table 3) and clinical (Table 2) studies, DNA vaccines can delay tumor growth and elicit a strong immune response, especially an antigen-specific CTL response, but are rarely able to completely reject the tumor. These modest gains were reached by optimizing DNA vaccines in several aspects, such as plasmid design and delivery and administration strategies [1, 9, 37, 91] However, DNA vaccines alone are not able to overcome the tumor immune escape caused by the natural selection of tumor cell clones lacking immunogenic antigens or by immunosuppressive cells that are recruited to the TME (MDSCs, Tregs among others), which lead to the exhaustion of T effector cells [7]. Cancer DNA vaccines can reach their optimum efficacy if combined with other strategies that can not only potentiate the antigen response but also silence immunosuppression in the TME [92].

There is evidence that combining therapeutic cancer vaccines with traditional modalities (radiotherapy, chemotherapy, surgical removal) may be synergistic. The combination therapies already tested in clinical and preclinical studies can be summarized as follows:

#### Cytokines/adjuvants

Immunostimulatory cytokines can increase the effect of the vaccine on effector T cells. They are generally encoded by the antigen-encoding vaccine, by another plasmid or injected as proteins in combination with the vaccine. In recent studies, the most commonly used cytokines include IL-2, IL-12 and GM-CSF. IL-2 is involved in the differentiation of immature T cells into both Tregs and effector T cells. Its great efficacy against metastatic melanoma and metastatic renal cell carcinoma led to its approval by the FDA [7, 93]. IL-12 is another important cytokine involved in T cell activation and effector function, and its combination with a vaccine increases the vaccine’s efficacy [94]. A plasmid encoding IL-12 combined with a DNA vaccine against cervical cancer promoted mouse survival and decreased the number of MDSCs in the TME [95]. GM-CSF is used in many clinical trials (Table 2) for its activity on DC maturation and T cell activation and proliferation. However, this molecule can also attract MDSCs, and it is not clear how this cytokine balances between immune activation and inhibition in vivo. Current clinical studies are seeking to answer this question [7]. Other cytokines could be used in combination with DNA vaccines, e.g., INFγ, IL-15, and IL-7 [7, 9].

The combination with other types of adjuvants could also be tested, such as TLR-activators. Recently, we demonstrated that the insertion of some CpG immunostimulatory motifs inside the antigen gene sequence through codon optimization could enhance cytokine production, thus increasing the efficacy of a DNA vaccine against P815 mastocytoma [37].

#### Immune checkpoint blockade (ICB)

The signaling mechanism mediated by costimulatory/inhibitory molecules plays an important role in T cell-mediated immunity. Many cells in the TME can express ligands for inhibitory receptors on T cells, leading to their inactivation [96]. Inhibitory receptors include CTLA-4, PD-1, TIM-3, LAG-3, etc. [96] In several studies, the in vivo blockade of CTLA-4 delayed tumor growth in animal models and resulted in tumor rejection in patients affected by melanoma [97, 98]. This effect was mainly due to the inhibition of TGF-β- and IL-10-secreting Tregs and an increased T effector cell activation [99]. Interestingly, this also resulted in immunity against the secondary exposure to tumor cells, suggesting that the memory component of the immune response can be evoked by anti CTLA-4 antibodies [100]. Antagonist antibodies that target PD-1 and its ligand PD-L1 have also achieved impressive and durable results in many solid tumors, leading to their FDA approval for different cancer types [7]. Recently, a relationship between ICB administration and the neoantigen burden has been demonstrated [101]. Snyder et al. sequenced 64 patients with advanced melanoma and showed that the somatic mutation burden was strongly associated with the clinical response to anti-CTLA4 [102]. Similarly, Rizvi et al. demonstrated that the mutation burden was a strong predictor of clinical response in non-small cell lung cancer (NSCLC) patients treated with anti-PD1 therapy, and that this therapy enhances neoantigen-specific T cell reactivity [103]. The higher prevalence of somatic mutations in cancer cell genomes was a common feature among cancers with a higher probability of responding to ICB. Thereafter, the link between the mutation burden and the clinical benefit following ICB immunotherapy was validated multiple times and in multiple tumor types [63]. This is related to the concept that with an increased tumor mutation burden, the probability of a cognate T cell clonally expanding against a specific tumor antigen will increase. In other words, high tumor mutation burden tumors often have more neoantigens that could be recognized by the processes involved in antitumor immunity, making such cancers more likely to respond to ICB therapy [59, 104].

To increase T cell activity in the TME and to broaden the number of patients responding to ICB, combinations of ICB with different strategies were tested for a variety of malignancies in preclinical and clinical studies [96]. Some examples include combination with radiation therapy [105], other antibodies [106], photodynamic therapy [107], and cancer vaccines [108]. Combination with cancer DNA vaccination seems to be promising in coupling the benefits of ICB with the ability of vaccines to prime the antigen-specific CTL response [88, 109]. A potent cancer vaccine that induces a T cell response against tumor-specific antigens could also increase the number of responders to ICB [63]. However, only a minority of patients respond to ICB therapy, suggesting the need for a rational use of ICB based on biomarkers predictive of the immune response to avoid nonresponsiveness to therapy and undesired side effects [110, 111].

#### Chemotherapy/targeted therapy

In the last few years, it has been reported that anticancer chemotherapy can play a double role in tumor eradication. Many chemotherapeutic drugs, such as gemcitabine [112], paclitaxel [113], cyclophosphamide [114] and others, applied in ultralow (metronomic) noncytotoxic doses, not only target tumor cells inducing TA release but also enhance T cell infiltration/activity in the TME and remove immunosuppressive cells. In a preclinical study, the combination of cyclophosphamide with DNA vaccines enhanced mouse survival and decreased the expression of immunosuppressive cytokines, such as IL-10 and VEGF [115]. Based on preclinical and clinical studies, the combination of the appropriate chemotherapeutic drug and vaccine therapy may play a substantial role in future cancer treatments, especially when patients do not respond to ICB [116]. Indeed, it has been demonstrated that treatment with chemotherapy restored sensitivity to checkpoint blockade through TLR4 simulation [116]. Further clinical studies are necessary to better define the optimal agents and schedule of administration.

DNA vaccines could also be combined with targeted therapies that are able to mediate tumor cell antigen release and enhance T cell priming. Sunitinib, a multitargeted receptor tyrosine kinase inhibitor, was found to decrease Tregs and MDSCs and increase INFγ-producing T cells in renal cell carcinoma patients [117]. The combination of sunitinib with a viral vaccine encoding CEA decreased the tumor volume in a mouse model [118]. Although not already tested with DNA vaccines, other tyrosine kinase inhibitors already approved by FDA, such as pazopanib, axitinib, and cabozantinib, could improve the patient response to vaccination.

#### Combination with other therapies

Other strategies that can be used in combination with DNA vaccines include endocrine therapy and radiotherapy (RT).

In hormonally driven tumors such as prostate cancer and breast cancer, endocrine therapy is part of the standard of care, and the effect of letrozole in decreasing the Tregs in the TME has already been demonstrated [119]. Furthermore, androgen deprivation in prostate cancer induces thymic regeneration and increases the number of effector T cells [7]. In an ongoing clinical trial (NCT02204098, phase I), the effect of Mam-A vaccine administration in combination with anastrozole, letrozole, tamoxifen, exemestane, and goserelin is under investigation.

Preclinical data have demonstrated the additive effect of RT and vaccines with an enhanced destruction of tumor cells, the release of TAs, an increase in IFNg production, and a global decrease of the tumor volume. T cells specific for other antigens not included in the vaccine were also generated [120, 121]. Since RT is a part of the standard of care, many trials using vaccines after radiation try to evaluate the mutual effects from the 2 therapies (Table 2).

**Fig. 3:**
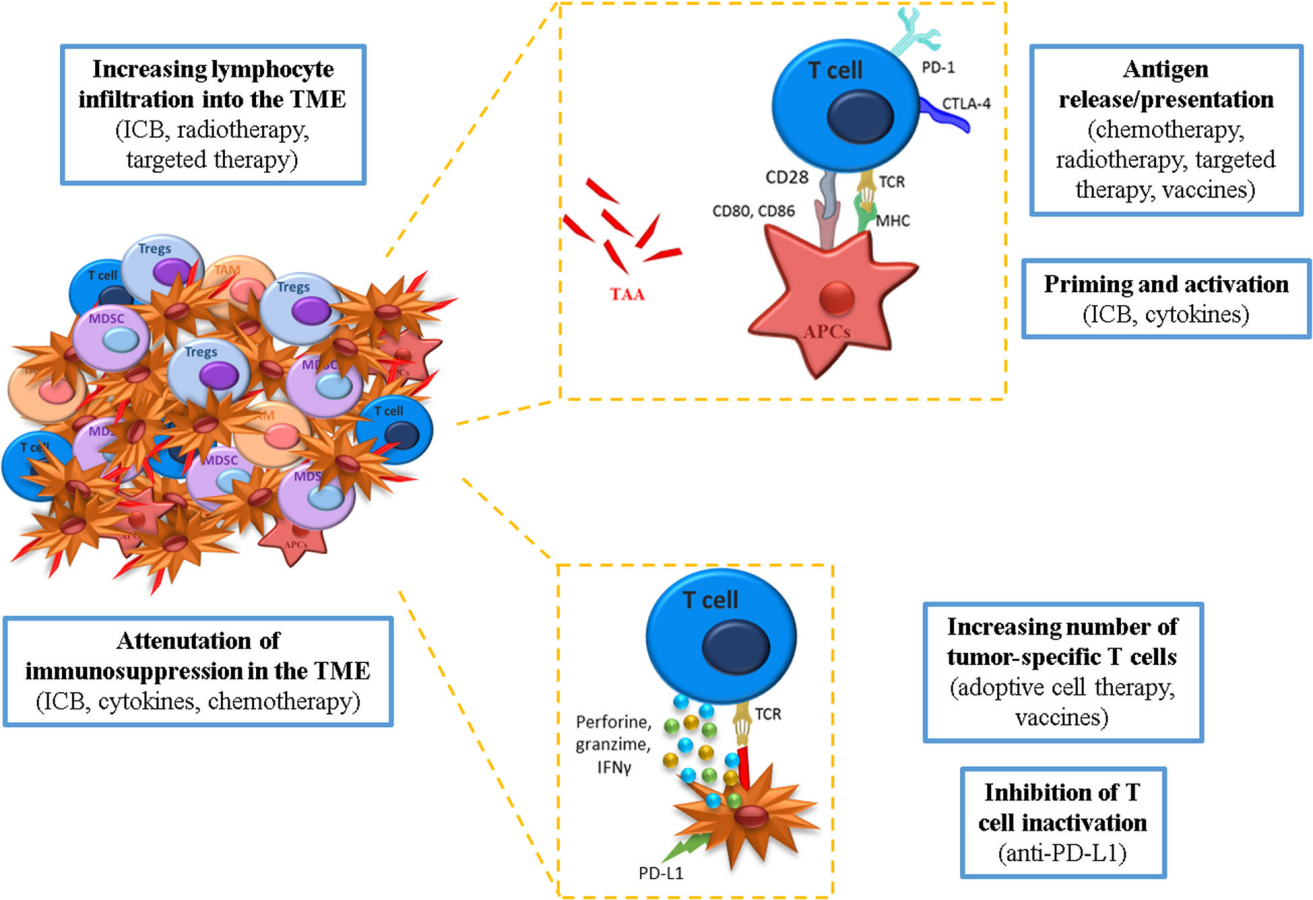
Mechanisms of action of therapies postulated to mediate synergistic effects in combination with DNA

In Fig. 3, the main mechanisms of action of therapies postulated to mediate synergistic effects in combination with DNA vaccines are shown.

### Results of completed clinical trials

Many already completed clinical trials tested the efficacy of DNA vaccines against different tumor types, such as breast, cervical, pancreatic and prostate cancers, multiple myeloma, and melanoma. These trials aimed at principally evaluating the safety and immunological response of DNA vaccines. A search for studies with “cancer” and “DNA vaccines” in clinicaltrials.gov [122] revealed 48 studies in the last 10 years with the following criteria: “completed”, “suspended” and “terminated”. Among the trials using DNA vaccines in a therapeutic approach, only a few of them have published results to date. Here, a non-exhaustive list of completed studies using naked DNA vaccines and containing results is described.

The NCT01304524 phase IIb clinical study tested the safety and efficacy of VGX-3100, a DNA vaccine targeting HPV 16 and 18 E6 and E7 proteins for cervical intraepithelial neoplasia grade 2/3. Six milligrams of the vaccine were delivered by IM EP at 0, 4 and 12 weeks without any severe side effects, but only mild reactions in the injection site, fatigue, nausea and general malaise in some of the patients. The vaccine was generally well tolerated and showed great efficacy against the pathology in almost 50% of the treated patients, as shown in the histopathological and immunological analysis. Indeed, VGX-3100 elicited significantly increased frequencies of antigen-specific activated CD8+ T cells and a higher humoral response compared to the placebo, making it the first therapeutic vaccine to elicit a complete adaptive immune response in patients with preinvasive cervical disease caused by HPV-16 and 18 [123]. Two phase III clinical trials (NCT03185013 and NCT03721978) using VGX-3100 are ongoing, as shown in Table 2.

Recently, Kim et al. published the results of the clinical trial NCT01634503 concerning the safety and efficacy of GX-188E, another plasmid DNA encoding the E6 and E7 proteins of HPV serotypes 16 and 18. The vaccine was injected 3 times (weeks 0, 4 and 12) IM to alternating deltoid muscles, and three different doses were tested (1, 2 or 4 mg). Importantly, 8/9 of the patients exhibited an enhanced polyfunctional HPV-specific CD8 T cell response, and 7/9 of the patients displayed a complete regression of their lesions and viral clearance within 36 weeks of follow-up. The vaccine administration did not elicit serious vaccine-associated adverse events and was estimated to be safe and well tolerated [124].

Other published results show the properties of mammaglobin-A (Mam-A) DNA vaccination for patients with breast cancer. Mam-A is a tumor-specific secretory protein overexpressed in 80% of human breast cancers. In a phase I clinical trial (NCT00807781), 4 mg of a pING-Mam-A DNA vaccine was administered at weeks 1, 4 and 8 IM to patients with metastatic breast cancer. The first results demonstrated the safety of the vaccine, with no significant side effects. The main observations about its efficacy were (i) an increase in the generation of specific Mam-A CD8+ T cells and IFN-γ production; (ii) a decrease in the frequency of Tregs and lower levels of IL-10; and (iii) an improved progression-free survival compared to the control group. These encouraging results suggest that Mam-A DNA vaccination can induce antitumor immunity in breast cancer patients and increase survival time [125–127].

In another phase I/II study (NCT00859729), 50–1600 μg of a pVAXrcPSAv531 plasmid coding for the full-length PSA protein were ID injected and electroporated in patients with relapsed prostate cancer. The vaccine followed radiotherapy and endocrine therapy with an LH-RH analogue (leuprorelin). No systemic toxicity was observed, and discomfort from EP did not require the use of topical anesthetics. A general increase in T cell reactivity was observed in most patients, although IM immunization seemed to result in more potent antibody responses [128].

A personalized DNA vaccine was tested in patients with multiple myeloma in a phase I clinical trial. The DNA encoded a patient-specific single chain variable fragment linked to fragment C of the tetanus toxin. Six doses of 1 mg of the vaccine were injected IM after chemotherapy or autologous stem cell transplant. In total, 72% of the patients generated a cell-specific immune response, and the overall survival was 64% after a median follow-up of 85.6 months [129].

A phase II clinical trial (NCT01334060) evaluated the safety and efficacy of a pDOM-WT1–37 and pDOM-WT1–126 DNA fusion gene vaccine encoding the Wilms tumor antigen 1 for leukemia patients. The plasmid was injected using IM EP, with no severe side effects. However, combination strategies to expand T cell responses with immunomodulatory antibodies are in development [130].

Interestingly, Niethammer et al. reported a phase I clinical trial (NCT01486329) using an oral vaccine (VXM01) against the VEGF-Receptor 2 with Salmonella typhimurium as a carrier, in addition to chemotherapy with gemcitabine, in patients with stage IV and locally advanced pancreatic cancer. The doses consisted of a solution containing 106, 108, 109, and 1010 colony forming units of VXM01. VXM01 represents a novel strategy by not targeting a tumor cell-resident antigen but instead targeting a tumor stroma-resident antigen overexpressed by the nonmalignant endothelial cells of the tumor neovasculature, giving the vaccine the potential to target many cancer types [131]. The same vaccine is also being tested in patients with glioblastoma (NCT02718443).

Another 19 studies were found in PubMed using the following criteria: “cancer DNA vaccine”, article type “clinical trial”, starting from 2013 until now. Most of the studies focus on prophylactic immunization with HPV DNA vaccines. Two phase I studies show some results of therapeutic cancer DNA vaccination (NCT00250419 and NCT00647114). Both of them used the HER2/CEA DNA vaccine V930 and showed the instauration of both humoral and cellular immune responses with no detectable immune response against the vaccine itself. As CEA and HER2 are expressed by many solid tumors, patients with different types of cancer were recruited. The vaccination dose was on the order of a few milligrams every 14 days for 5 injections, and the plasmid was injected by IM EP. However, in this case, no evidence of an increase of a HER/2 or CEA-specific response was observed [132].

Overall, vaccination is used after conventional therapies. Completed, terminated and suspended clinical trials reported only minor discomfort after vaccination, no important side effects and, generally, an increased number of CD8+ T cells specific for the antigen encoded by the DNA vaccine. Most of the trials used DNA vaccines encoding TAAs, and only a few tested personalized approaches.

### Ongoing human clinical trials using therapeutic cancer DNA vaccinations

In searching all the cancer DNA vaccine interventional clinical studies in the last 10 years with the criteria “not yet recruiting”, “recruiting”, “enrolling by invitation” and “active nonrecruiting”, we found 56 studies. Among them, 27 studies used DNA vaccines as naked plasmids not encapsulated in cells or in virus-like nanoparticles. These studies are listed in Table 2. They are all in clinical phase I or I/II or II, and DNA vaccines are generally administered after the standard of care for each cancer type, including surgical ablation, radiotherapy and/or chemotherapy. The results for these trials are not yet available, except for the trial NCT00849121. This study used a DNA vaccine encoding PAP, with GM-CSF as an adjuvant, administered ID into patients with prostate cancer. Only one of the 17 patients experienced a vaccine-related adverse event of grade 2 or more, more than half had a great PAP-specific CTL response, and in 7/17 patients, the PSA doubling time increased during the treatment period. Twelve of the 17 patients (70%) were metastasis-free after one year of treatment (clinicaltrials.gov).

Another study with the criteria “DNA electroporation” and “cancer” led to 3 more trials (“not yet recruiting”, “recruiting”, “enrolling by invitation” and “active nonrecruiting”) in the last 10 years: NCT03499795, NCT03491683, and NCT02301754. With the criteria “plasmid” and “tumor”, we found 2 additional studies: NCT02531425 and NCT03502785. These are all listed in Table 2.

Of particular interest are the only 2 studies we found in phase III (NCT03721978 and NCT03185013) using VGX-3100 delivered by IM EP against cervical cancer.

**Fig. 4:**
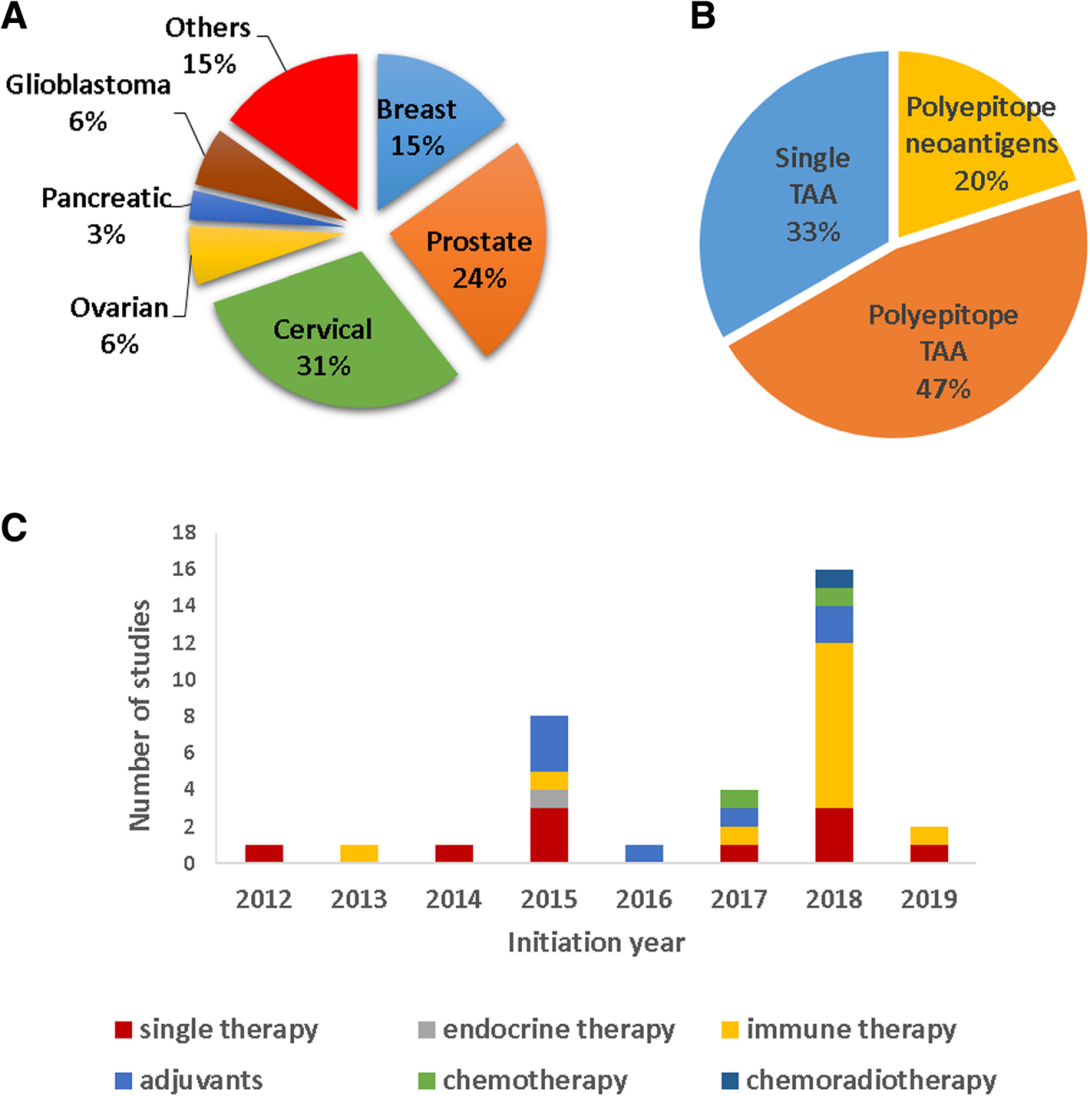
Ongoing clinical trials of the analyzed studies. **a** Cancer types using cancer DNA vaccines in clinical trials. **b** Type of antigens encoded in the DNA vaccine. **c** Studies combining cancer DNA vaccines with other therapies (endocrine therapy, immunotherapy, chemotherapy, chemoradiotherapy or adjuvants) or using DNA vaccines as a single therapy

Breast, prostate and cervical cancer are the most studied in the trials (Fig. 4a). Most of the vaccines encode well-known TAAs (E6/7 HPV protein for cervical cancer, Mam-A or HER2 for breast cancer, prostatic acid phosphatase (PAP) for prostate cancer, etc.). Only 17% of the clinical trials used personalized/neoantigen vaccines (e.g., NCT02348320 and NCT03122106), as shown in Fig. 4b. This number has increased in recent years: 80% of the trials using neoantigens started in 2018–2019. Generally, more than one epitope is encoded by the DNA vaccines in both TAA and neoantigen vaccines (Fig. 4b).

DNA vaccines are mostly associated with other therapies: immunotherapies (antibodies anti-HER2, anti-CTLA4, anti-PD1, anti-PD-L1, and cell vaccines), immune adjuvants (GM-CSF, hIL-12, etc.) generally injected with the DNA vaccine or encoded in the vaccine itself, chemotherapy (carboplatin, paclitaxel, cyclophosphamide), and endocrine therapies (anastrozole, letrozole, tamoxifen, exemestane, and goserelin). In recent years, the number of studies using other therapies in combination with DNA vaccines has also increased (Fig. 4c). DNA vaccines are usually injected IM or ID, in rare cases SC or in the lesion/tumor, and electroporated after the injection. The doses can vary from 100 μg to a few mg. The regimen of administration depends on the type of vaccine, but in all trials, vaccines are injected more than once, at 2–4 weeks of intervals, and the therapy lasts for a few months.

### Current challenges and future perspectives

Past and ongoing clinical studies investigate DNA vaccines that are optimized using various strategies. The use of a codon-optimized, polyepitopic DNA vaccine encoding TAAs or neoantigens and their combination with other therapies to modulate the immunosuppressive TME seem to be the most relevant options. However, many questions still need to be addressed.

#### Selection of the encoded antigen(s)

The first question concerns the antigen type: TAAs or neoantigens? Relevant TAAs have been identified for most tumors, but immune tolerance can limit their efficiency. On the other hand, neoantigen identification is time consuming and expensive, and neoantigens do not reflect the tumor heterogeneity in the individual patient (e.g., in metastasis). Second, how many antigens should be encoded in the same plasmid? It is still not clear whether one antigen is superior to another in terms of frequency of immune response or clinical effect [133]. In one study, it has been shown that neoantigens with a predicted high affinity are more immunogenic and that a poly-specific and poly-functional DNA vaccine encoding neoantigens was the most efficient solution to prevent tumor growth in mice [134]. Further studies are needed to generalize these findings. Most of the clinical trials use a relevant antigen for the vaccination (e.g., PAP, E6/E7), but none compare the combination of this antigen with others or with neoantigens to evaluate their effect on immune activation. This point arouses a last question: is it worth mixing TAAs and neoantigens in the same vaccine? Furthermore, should the different antigens be administered in the same plasmid or is it better to encode them in different plasmids administered together? To our knowledge, none of the clinical studies address this question, which could be a further area of investigation in the future.

#### Selection of the combination therapy and treatment schedule

An important parameter in the combinatorial approach is the choice of the right therapies to be combined. This aspect depends on the tumor type, its presence, and the possibility of detecting patient-specific biomarkers, among others. In addition, the administration schedule of multiple therapies is crucial. Until now, vaccines have been used as a last-line therapy. To integrate them as a standard of care therapy, it is crucial to know when to administer them. This decision should consider the time for the immune system to generate a specific immune response against the delivered antigen, the need for multiple doses of administration and the interaction with the combined therapy. For example, some studies revealed that using ICB after the peptide/RNA vaccine treatment induced a sustained remission with no sign of disease recurrence [66]. However, this should be adapted to the specific therapy and patient. Furthermore, the doses should be consequently adapted to the combination regimen.

#### Find a good preclinical model

Most of the critical points aroused in cancer DNA vaccination can ultimately be addressed only in clinical practice because translation from animals is extremely difficult. This is due to the different tumor characteristics and the differences in the immune system between humans and animals [135]. To try to overcome this problem, many orthotopic injection models, genetically engineered mice, xenograph and humanized models have been developed. However, they all fail to recapitulate the chaotic way in which malignant transformation occurs during cancer development in human patients. Mouse models provide valuable insight into the mechanisms of action and provide important proof of concept for human studies, but there remains a need for larger animal models encompassing a fully competent immune system. Some researchers suggest the use of canine and porcine models, especially for skin cancers [135]. However, housing, ethical regulation, and breeding difficulties limit the use of big animal models. Furthermore, even these models have limitations, and the idea of a universal model for oncoimmunology currently seems unrealistic.

#### The variability problem: the necessity of biomarkers and therapy standardization; how far from therapy personalization?

Immunotherapies have a variable response rate from one patient to another and are often associated with side effects. For this reason, it is important to identify biomarkers that could predict the patient response to a specific therapy and to standardize the therapy according to the predicted biomarker. Furthermore, biomarkers may be useful for monitoring treatment response. However, the translation of biological data into predictive or prognostic biomarkers is complicated by the complex interactions between tumors and the immune system and by host and tumor variability. Many studies are using bioinformatics tools and new genomic and proteomic technologies to predict specific tumor signatures, generating complex datasets that give rise to analytical challenges. Currently, we can rely on imperfect biomarkers, such as PD-L1 expression in the tumor or the tumor mutation burden. This gap in knowledge leaves room for further studies that will help treatment selection and design the best combination therapy for each patient.

## Conclusions

The analysis of recent preclinical and clinical trials suggests that the current therapeutic cancer vaccines are unlikely to dramatically impact cancer outcomes as a single agent. Many combinations with other strategies have been tested, demonstrating the greater potential of the combination on improving clinical outcomes compared to the single therapy. The personalized approaches both in the vaccine design and in the choice of combination therapy will be crucial for success in the clinic. Furthermore, since DNA vaccines are well tolerated and safe, their combination with other therapies could become part of the standard of care in many malignancies.

We anticipate that, in the future, personalization in the DNA vaccine design will be coupled with personalization in the choice of the most appropriate combined therapy, following the analysis of single patient specificity and biomarkers that can predict the response to a specific agent. This could represent the best approach to increase the efficacy of cancer immunotherapy and reduce the adverse effects linked to a nonspecific treatment.

## Abbreviations

CAR: Chimeric antigen receptor
CTL: Cytotoxic T cell
ICB: Immune checkpoint blockade
ID: Intradermal
IM: Intramuscular
MDSC: Myeloid-derived T cell
MHC: Major histocompatibility complex
PADRE: Pan DR *epitope*
*RT*: *Radiotherapy*
SC: Subcutaneous
TA: Tumor antigen
TAA: Tumor-associated antigen
TAM: Tumor-associated macrophages
Th: T helper
TME: Tumor microenvironment
Treg: Regulatory T cell
TSA: Tumor-specific antigen
